# Efficacy and safety of Shengxian Yixin Granule for treating heart failure with preserved ejection fraction: a study protocol for a multicenter, randomized, double-blind, placebo-controlled trial

**DOI:** 10.3389/fcvm.2026.1902637

**Published:** 2026-08-13

**Authors:** Xiaozhen Cheng, Runhan Ma, Mengsi Guo, Jingjing Wei, Qifei Zhao, Rui Yu, Zhengwei Dong, Janru Wang, Yongxia Wang, Mingjun Zhu, Xinlu Wang

**Affiliations:** 1 Department of Cardiovascular Disease, The First Affiliated Hospital of Henan University of Chinese Medicine, Zhengzhou, China; 2 Collaborative Innovation Center of Prevention and Treatment of Major Diseases by Chinese and Western Medicine, Zhengzhou, Henan, China; 3 First School of Clinical Medicine, Henan University of Chinese Medicine, Zhengzhou, China

**Keywords:** effectiveness, heart failure with preserved ejection fraction, randomized controlled trial, safety, Shengxian Yixin Granule, study protocol

## Abstract

**Introduction:**

Heart failure with preserved ejection fraction (HFpEF) is a highly complex clinical syndrome associated with significant morbidity and mortality. Its underlying mechanisms are poorly understood, treatment options are limited, the disease burden on patients is substantial, and clinical outcomes are poor. Previous clinical studies have preliminarily suggested that Shengxian Yixin Granule (SXYX) is an effective herbal formula for the treatment of HFpEF, but it lacks high-quality clinical evidence. This study therefore plans to conduct a randomized controlled trial (RCT) to evaluate the efficacy and safety of SXYX in treating HFpEF, aiming to provide a new therapeutic strategy for this condition.

**Methods:**

This study is a multicenter, randomized, double-blind, placebo-controlled clinical trial. It will be conducted at four Chinese medicine hospitals in China. A total of 148 eligible patients with HFpEF will be enrolled and randomly assigned in a 1:1 ratio to the SXYX group or the placebo group. The SXYX group will receive Shengxian Yixin Granule, while the placebo group will receive a matching placebo. Both groups will receive standard Western medical therapy as background treatment. The treatment duration for all groups is 24 weeks. The primary outcome is the change in peak VO_2_ (assessed by cardiopulmonary exercise testing) from baseline to 24 weeks. Secondary outcome measures include the change in peak oxygen uptake from baseline to 12 weeks, NT-proBNP, echocardiographic parameters, NYHA functional class, 6-minute walk test distance (6MWT), Traditional Chinese Medicine (TCM) syndrome scale, major symptoms and signs, EQ-5D score, and coagulation function. These outcomes will be used to evaluate the efficacy of SXYX. Routine blood, urine, and stool tests, liver and kidney function tests, electrocardiograms, and adverse events will be used to assess the safety of SXYX. Wearable monitoring devices will be used for out-of-hospital, dynamic, and multidimensional assessment of patients’ exercise tolerance, including heart rate, blood oxygen saturation, step count, and walking distance.

**Discussion:**

This study will evaluate the efficacy and safety of SXYX in treating HFpEF. The findings will provide a scientific basis for the clinical application of SXYX.

**Clinical Trial Registration:**

https://itmctr.ccebtcm.org.cn, identifier ITMCTR2026000833.

## 1 Introduction

Heart failure affects more than 64 million people worldwide. With population aging and improvements in post-myocardial infarction treatment and survival rates, its prevalence continues to rise. Among the various subtypes, the prevalence of HFpEF has increased significantly in recent years (1, 2). HFpEF is a complex clinical syndrome characterized by a left ventricular ejection fraction (LVEF) ≥50%, along with symptoms such as dyspnea and reduced exercise tolerance. HFpEF accounts for approximately 50% of all heart failure cases and has become the predominant form of heart failure worldwide (3, 4). HFpEF is associated with substantial morbidity and mortality, with a 30-day readmission rate of approximately 20%. In-hospital, 1-year, and 5-year mortality rates range from 2.4% to 4.9%, 20% to 29%, and 53% to 74% (5, 6). Landmark studies in the treatment of HFpEF include the EMPEROR-Preserved and DELIVER trials, which demonstrated that SGLT2 inhibitors reduced the composite endpoint of cardiovascular death or hospitalization for heart failure (7, 8). However, the absolute risk reduction for the primary composite endpoint in these trials was approximately 3%, and this benefit was largely driven by a reduction in heart failure hospitalizations or urgent visits, rather than a decrease in cardiovascular mortality (9). STEP-HFpEF and STEP-HFpEF DM trials demonstrated that in patients with HFpEF and comorbid obesity (with or without type 2 diabetes), treatment with the GLP-1 receptor agonist semaglutide significantly improved heart failure-related symptoms, enhanced exercise tolerance, and substantially reduced body weight (10, 11). The SUMMIT trial further showed that in patients with HFpEF and obesity, the GIP/GLP-1 dual receptor agonist tirzepatide reduced the risk of the composite endpoint of cardiovascular death or worsening heart failure and improved health status (12). However, these benefits were observed only in HFpEF patients with comorbid obesity. In the PARAGON-HF trial, sacubitril/valsartan did not achieve a statistically significant reduction in the primary composite endpoint of total heart failure hospitalizations and cardiovascular death among the overall HFpEF population (13). Despite significant advances in the prevention and management of HFpEF in modern medicine, patients’ treatment needs remain unmet.

Traditional Chinese Medicine (TCM) has been used for thousands of years and is widely applied in clinical practice for the treatment of acute and chronic heart failure, which has shown promising but preliminary evidence of efficacy, although the existing studies are limited by heterogeneity in study design, patient populations, and TCM formulations. TCM treatment for HFpEF has been shown to significantly improve clinical symptoms, enhance quality of life, and increase exercise tolerance, as well as to reduce fluid retention, ameliorate cardiac remodeling, and improve cardiac function (14, 15). Multiple meta-analyses of RCTs have shown that integrated traditional Chinese and Western medicine therapy improves quality of life and exercise tolerance in HFpEF patients and has a favorable safety profile (16, 17). According to the *2025 Expert Consensus on the Integrated Traditional Chinese and Western Medicine Diagnosis and Treatment of Heart Failure with Preserved Ejection Fraction* (18), the main TCM syndrome patterns of HFpEF include Qi deficiency with blood stasis, Qi-Yin deficiency with blood stasis, and Yang deficiency with blood stasis and fluid retention, among which Qi deficiency with blood stasis is the most common type in clinical practice. Such patients present with Qi deficiency manifestations, including fatigue, reluctance to speak, easy fatigability upon exertion, spontaneous sweating, and a low, weak voice, as well as blood stasis manifestations, such as a purplish-dark complexion, lips, or nails, and a pale-dark tongue with petechiae or ecchymoses.

Our team developed SXYX based on the core pathogenesis of Qi deficiency with blood stasis in HFpEF patients, following the therapeutic principle of supplementing Qi and elevating sunken Qi, as well as activating blood circulation and resolving stasis. SXYX is widely used for the treatment of HFpEF patients at The First Affiliated Hospital of Henan University of Chinese Medicine as a hospital-approved formula that can be prescribed at any time (Yu Pharmaceutical Preparation Approval No. Z20250008000, where “Yu” is the official abbreviation for Henan Province). It has also been granted a China Invention Patent (Patent No. ZL202310555277.X). SXYX consists of 13 herbal medicines (see Table 1). Preliminary clinical studies have shown that SXYX is safe and effective for treating HFpEF; however, these earlier studies had limitations, including a lack of scientific rigor in study design and an insufficient sample size. Therefore, the present study was designed as a multicentre, randomised, double-blind, placebo-controlled clinical trial, incorporating data from wearable monitoring devices. The study aims to systematically evaluate the clinical efficacy and safety of SXYX in treating HFpEF, and to provide a scientific basis for the clinical application of SXYX.

**Table 1:** The composition of Shengxian Yixin Granule.

Chinese name	Latin name	Medicinal part used	Weight ratio
Huang Qi	Hedysarum Multijugum Maxim	Root	30
Dang Shen	Codonopsis Radix	Root	20
Zhi Mu	Anemarrhenae Rhizoma	Rhizome	10
Chai Hu	Radix Bupleuri	Root	6
Sheng Ma	Cimicifugae Rhizoma	Rhizome	6
E Zhu	Curcumae Rhizoma	Rhizome	15
Gui Zhi	Cinnamomi Ramulus	Tender Twig	9
Gua Lou	Trichosanthes Kirilowii Maxim	Fruit	20
Xie Bai	Allium Azureum Ledeb	Bulb	20
Ban Xia	Arum Ternatum Thunb.	Tuber	15
Chi Shao	Radix Paeoniae Rubra	Root	15
Ting Li Zi	Lepidii Semen Descurainiae Semen	Seed	15
Gan Cao	licorice	Root and Rhizome	12

The exact daily dosage of individual herbs is not disclosed due to proprietary protection. The composition is presented as weight ratios (relative proportions) based on the raw herb materials.

## 2 Methods and analysis

### 2.1 Study design

**Figure 1:**
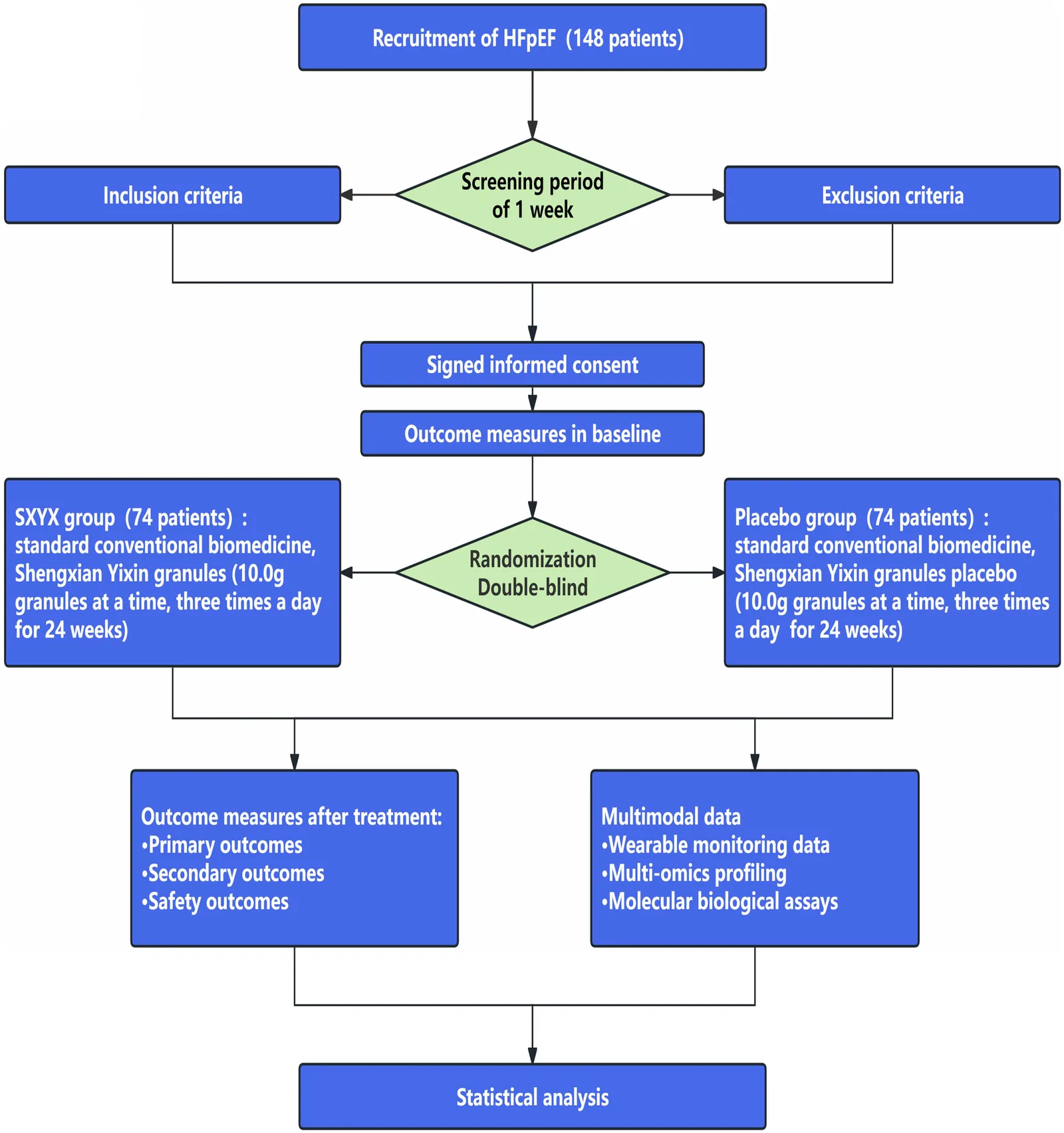
Flow chart of the study design. HFpEF, heart failure with preserved ejection fraction; SXYX, Shengxian Yixin Granule.

This study is a multicenter, randomized, double-blind, placebo-controlled, parallel-group clinical trial. The study protocol has been approved by the Ethics Committee of The First Affiliated Hospital of Henan University of Chinese Medicine (approval No. 2026HL-041-01). The trial was registered on 27 March 2026 with the International Traditional Medicine Clinical Trial Registry (ITMCTR) at https://itmctr.ccebtcm.org.cn (registration number: ITMCTR2026000833). The study protocol was developed in strict accordance with the SPIRIT 2025 guidelines (19). The results of this trial will be reported in accordance with the CONSORT 2025 statement (20). The study will be conducted at four TCM hospitals in China: The First Affiliated Hospital of Henan University of Chinese Medicine, Lushi County Hospital of Traditional Chinese Medicine, Fangcheng County Hospital of Traditional Chinese Medicine, and Mianchi County Hospital of Traditional Chinese Medicine. A total of 148 eligible patients with HFpEF meeting the inclusion and exclusion criteria will be enrolled in the study. After providing written informed consent, patients will be randomly assigned in a 1:1 ratio to receive either SXYX or placebo for 24 weeks of treatment. The trial flow diagram is shown in Figure 1.

### 2.2 Sample size calculation

This study is a multicenter, randomized, double-blind, placebo-controlled trial. The primary outcome is a continuous variable. The allocation ratio between the SXYX group and the placebo group is 1:1. The sample size will be calculated using the formula for comparing two independent means. The formula is as follows:

$$n_{1}=n_{2}=2{\left[\frac{(Z_{\alpha /2}+Z_{\beta })\sigma }{\delta }\right]}^{2}$$

Based on preliminary data from a small-sample, exploratory pilot study, the peak oxygen consumption (VO_2_ peak) in patients with HFpEF after 6 months of treatment was 20.2 ± 4.8 mL/kg/min in those receiving Chinese herbal medicine, and 18.9 ± 4.2 mL/kg/min in those receiving standard conventional medical therapy. The standard deviations of 4.8 and 4.2 from the preliminary data were used solely to estimate the population standard deviation (*σ*). Based on the OptimEx-Clin Study (21), the latest prognostic studies of percentage of predicted peak VO_2_ in HFpEF (22), and the published results of traditional Chinese medicine for HFpEF with the Qi-deficiency and blood-stasis pattern (23), the minimal clinically important difference for sample size calculation was set at 2.7 mL/kg/min.

$$\sigma^{2}=(S_{{}_{1}}^{2}+S_{2}^{2})/2=(4.8^{2}+4.2^{2})/2=20.34$$

$$n_{1}=n_{2}=2{\left[\frac{(Z_{\alpha }+Z_{\beta })\sigma }{\delta }\right]}^{2}=2\left[\frac{{\left(1.96+1.282\right)}^{2}\times 20.34}{2.7^{2}}\right]\approx 59$$

The calculation yielded approximately 59 patients per group. Accounting for an anticipated 20% dropout rate, the required sample size was adjusted to 74 patients per group, resulting in a total enrollment of 148 patients.

### 2.3 Inclusion criteria

**Figure 2:**
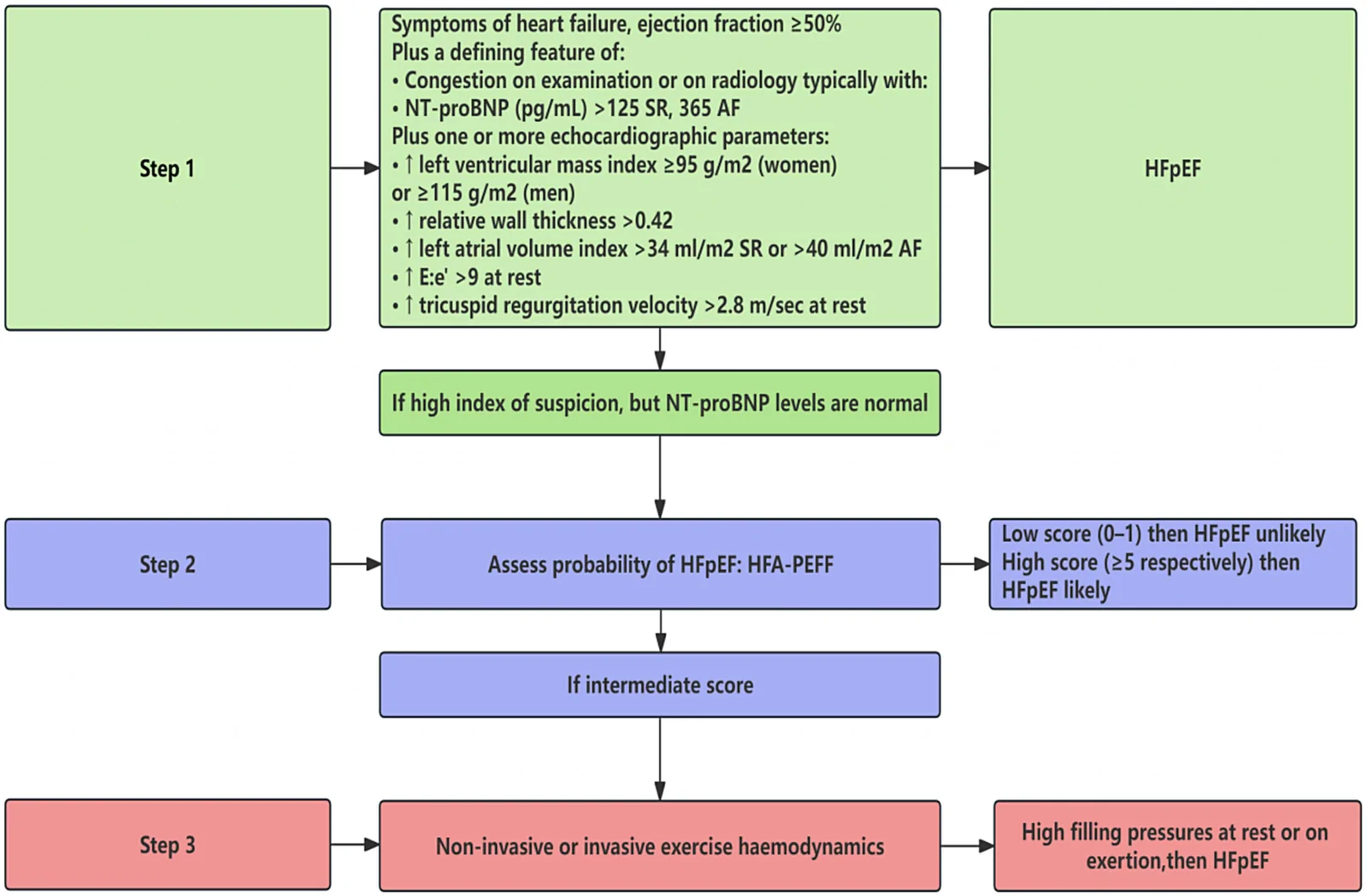
HFpEF diagnostic algorithm. AF, atrial fibrillation; E:e', early diastolic mitral inflow velocity to early diastolic mitral annulus velocity; HFA-PEFF, Heart Failure Association Pre-test assessment, Echocardiography and natriuretic peptide, Functional testing, Final aetiology; HFpEF, heart failure with preserved ejection fraction; NT-proBNP, N-terminal pro-B type natriuretic peptide; SR, sinus rhythm. Reproduced with permission from “HFpEF diagnostic algorithm” by Patricia Campbell, Frans H Rutten, Matthew MY Lee, Nathaniel M Hawkins and Mark C Petrie.

1. Aged 18–75 years, of any gender.
2. Based on the 2024 *Lancet Seminar “Heart failure with preserved ejection fraction: everything the clinician needs to know”* (3) and the 2019 *European Heart Journal consensus recommendation “How to diagnose heart failure with preserved ejection fraction: the HFA-PEFF diagnostic algorithm: a consensus recommendation from the Heart Failure Association (HFA) of the European Society of Cardiology (ESC)”* (24), the diagnostic criteria for HFpEF were jointly established. The pre-specified diagnostic threshold was set at ≥5 points for the confirmation of HFpEF. All echocardiographic recordings from the different sub-centres were sent to the central research laboratory for unified review. The diagnostic flowchart is shown in Figure 2.
3. Based on the *Guideline for Diagnosis and Treatment of Chronic Heart Failure in Traditional Chinese Medicine (2022)* (25) published in the Journal of Traditional Chinese Medicine in 2022, the diagnostic criteria for the “Qi deficiency and blood stasis pattern” of HFpEF were formulated. The primary symptoms include palpitations, wheezing, fatigue, and shortness of breath. The secondary symptoms are: (i) languidness, disinclination to speak, and easy fatigue with activity; (ii) spontaneous sweating without obvious cause during the day, aggravated after activity; (iii) dull purplish complexion; (iiii) dull purplish lips. For tongue and pulse: the tongue body is purplish (or with ecchymoses, petechiae, or tortuous and cyanotic sublingual collaterals), and the pulse is deep, thready, or feeble and forceless. A diagnosis can be made when two primary symptoms and two secondary symptoms are present, in combination with the tongue and pulse findings. The Traditional Chinese Medicine syndrome score scale is shown in the Table 2.
4. Patients were required to meet the following clinical stability criteria: New York Heart Association (NYHA) functional class II–III; no hospitalisation for acute heart failure, no acute coronary syndrome, and no percutaneous coronary intervention (PCI) or coronary artery bypass grafting (CABG) within the preceding 4 weeks; resting systolic blood pressure maintained within the range of 100–160 mmHg; active myocardial ischaemia rigorously excluded by resting electrocardiography, stress testing, or coronary computed tomography angiography; and a respiratory exchange ratio (RER) of ≥1.10 confirmed on cardiopulmonary exercise testing. In addition, patients’ symptoms and standard conventional medical therapy were required to be stable for at least 2–4 weeks before enrolment.
5. Patients who voluntarily provide written informed consent.

**Table 2:** Traditional Chinese medicine syndrome score scale (Qi deficiency and blood stasis pattern).

Symptoms		Scoring criteria
Primary symptoms	Palpitations	□0: None of the above symptoms □1: During ordinary physical activity □2: After slight exertion □3: Often occurs at rest
	Wheezing	□0: None of the above symptoms □1: During ordinary physical activity □2: After slight exertion □3: Often occurs at rest
	Fatigue	□0: None of the above symptoms □1: During daily activities □2: During mild exertion □3: Occurs without activity
	Shortness of breath	□0: None of the above symptoms □1: During daily activities □2: During mild exertion □3: Occurs without activity
Secondary symptoms	Languidness and disinclination to speak	□0: None of the above symptoms □1: Spiritlessness, fatigue, or shortness of breath with disinclination to speak □2: Low and weak voice or weak cough □3: Lassitude and drowsiness
	Spontaneous sweating	□0: None of the above symptoms □1: Slight sweating after activity □2: Moist skin, aggravated after slight exertion. □3: Spontaneous sweating even at rest, and profuse sweating on exertion, like being drenched.
	Complexion	□0: None of the above symptoms □1: Dull complexion without lustre □2: Sallow and dull complexion □3: Purplish-dark complexion
	Lip colour	□0: None of the above symptoms □1: Slightly dark lips □2: Purplish-dark lips □3: Purplish-black lips
	Tongue body	□0: None of the above symptoms □1: Pale and dark tongue body □2: Purplish tongue body □3: Purplish tongue body with scattered ecchymoses and petechiae, or tortuous and cyanotic sublingual veins
	Pulse condition	□0: None of the above symptoms □2: Deep and thready pulse, or feeble and forceless pulse

### 2.4 Exclusion criteria

1. A diagnosis of hypertrophic obstructive cardiomyopathy (HOCM), cardiac amyloidosis, hemochromatosis, Fabry disease, stress (Takotsubo) cardiomyopathy, constrictive pericarditis, muscular dystrophy, severe valvular heart disease, or Unstable coronary artery disease.
2. A diagnosis of stroke or transient ischemic attack (TIA) within the past 3 months, or having undergone coronary artery bypass grafting (CABG), valve replacement surgery, or other major cardiovascular surgery within the past 3 months.
3. Inability to exercise due to lung disease [e.g., forced expiratory volume in 1 s (FEV1) <50% of predicted value, or COPD GOLD stage III–IV], or any condition that may interfere with cardiopulmonary exercise testing.
4. Pregnancy, current breastfeeding, or planning to become pregnant during the study period.
5. Any serious illness that may affect the subject's clinical course, such as: Acute exacerbation of chronic obstructive pulmonary disease (COPD); Acute or chronic liver disease [alanine aminotransferase (ALT) ≥3× the upper limit of normal (ULN)]; Severe renal impairment [defined as serum creatinine (Cr) ≥3 mg/dL, estimated glomerular filtration rate (eGFR) ≤30 mL/min/1.73 m^2^, or receiving regular dialysis]; Anemia [hemoglobin (Hb) <90 g/L]; Gastrointestinal surgery or disease that may affect the absorption of the investigational drug; Acute or chronic pancreatitis; Severe primary hematologic or psychiatric disorders; Any documented active or suspected malignancy, or history of malignancy, within 1 year prior to screening.
6. Any disease (other than heart failure) that, in the investigator's judgment, is associated with a life expectancy of less than 6 months.
7. Known history of allergies, including allergies to multiple drugs or foods, or known allergy to any component of Shengxian Yixin Granule.
8. Inability to exercise, or conditions that, in the investigator's judgment, may affect exercise, inability to perform activities of daily living, or inability to take oral medications.
9. Use of herbal medicines with similar therapeutic effects (e.g., decoctions, patent herbal medicines, or herbal injections) within the past 7 days.
10. Receipt of any investigational therapy other than the study drug.
11. Suspected or documented history of alcohol or drug abuse.
12. Inability to comply with the study assessment schedule for follow-up visits, or judged by the investigator to be unsuitable for continued participation in the trial.

### 2.5 Withdrawal criteria and management

Regardless of the time or reason for withdrawal, any participant who does not complete the full course of the trial is considered a dropout. The investigator must document the reason for withdrawal in the case report form (CRF). The investigator should make every effort to contact the patient, complete as many of the scheduled assessments as possible, fill in the end-of-treatment follow-up record form, and document the time of the last dose whenever possible. For withdrawals due to adverse events, if after follow-up the event is ultimately judged to be related to the investigational drug, this must be documented in the CRF, the sponsor must be notified, and the event should be reported to the ethics committee as required.

### 2.6 Discontinuation criteria

(1) The subject is judged by the principal investigator to be unsuitable to continue in the trial; (2) a serious adverse event directly related to the study treatment occurs, prompting immediate discontinuation of the study treatment; (3) the patient has poor compliance and is unable to complete the treatment or efficacy assessments in accordance with the study protocol.

### 2.7 Termination criteria

(1) A major flaw in the trial protocol is identified during the trial, or significant deviations occur in its implementation; (2) the sponsor requests termination (e.g., due to funding issues, administrative reasons, etc.); (3) the regulatory authority requires termination.

### 2.8 Laboratory thresholds and adverse event management measures

Laboratory abnormalities will be graded and recorded according to the Common Terminology Criteria for Adverse Events (CTCAE), Version 6.0, published by the US National Cancer Institute (NCI) (26). Any adverse events occurring during the trial, including subjective discomfort of subjects and laboratory test abnormalities, must be taken seriously, carefully analysed, and managed promptly to ensure subject safety. Should adverse events such as hepatic or renal dysfunction, bleeding risk, drug or food allergy, or gastrointestinal discomfort arise during the trial, appropriate measures—including symptomatic treatment, dose adjustment, treatment discontinuation, or observation—may be taken depending on the nature and severity of the event. Investigators are required to follow up all adverse events until they resolve to baseline, are assessed by the investigator as stable, or the subject is lost to follow-up or withdraws informed consent. All adverse events should be documented in the source medical records and the adverse event pages of the CRF to facilitate source data verification.

### 2.9 Serious adverse event reporting procedure

Any serious adverse event occurring during the trial must be reported immediately to the principal investigator, the institutional drug clinical trial office, the ethics committee, and the project team, with the completion of a “Serious Adverse Event Report Form”, while simultaneously notifying the other investigators involved in the trial.

### 2.10 Randomization and blinding

#### 2.10.1 Randomization

This study uses the central randomization system (Interactive Web Response System, IWRS) of The First Affiliated Hospital of Henan University of Chinese Medicine, employing stratified block randomization for enrollment. The study will be conducted at four TCM hospitals. Using study site as the stratification factor, patients will be randomly assigned in a 1:1 ratio to either the SXYX group or the placebo group (74 patients per group). The randomization list was generated by an independent statistician using the IWRS, producing randomization sequence numbers from 001 to 148, ensuring balanced sample sizes within each stratum. Randomization assignment will be implemented and managed using the EDC system (Electronic Data Capture) of the Evidence-Based Medicine Center of The First Affiliated Hospital of Henan University of Chinese Medicine. Investigators will obtain the investigational drug numbers for enrolled patients through this system. The IWRS is integrated with the EDC system in real time. The system automatically conceals the unallocated randomization sequence from the assignment results, thereby achieving allocation concealment.

#### 2.10.2 Blinding

1. Blinding Design: The randomization code list will be generated by the EDC system. Before the start of the clinical trial, an independent statistician will label the investigational drugs according to the drug numbers in the blinding list.
2. Routine Unblinding and Emergency Unblinding: After the completion of the study, unblinding can be performed in the system, and the blinding list will be exported and provided to the statistical analysts for analysis. Emergency unblinding is required under the following conditions: (i) occurrence of a serious adverse event (SAE); (ii) occurrence of a serious complication; (iii) worsening of symptoms requiring emergency intervention. When emergency unblinding is deemed necessary, it should be performed after obtaining written approval from the site principal investigator, who will then log into the IWRS to perform the emergency unblinding. The lead hospital must be notified within 24 h after unblinding. Participants who withdraw from the trial due to perceived lack of efficacy must not be unblinded.
3. Recording and handling of unblinding events: All unblinding events will be documented in detail, reported by treatment group, and reviewed periodically by the Data Safety Monitoring Board (DSMB). A formal assessment of blind integrity will be triggered only if the unblinding rate is unusually elevated or if there is a marked imbalance in the distribution of unblinding events between groups. Emergency unblinding events will be explicitly handled as “intercurrent events” within the framework of ICH E9(R1) (27), and multiple strategies will be employed in sensitivity analyses to evaluate their impact on the robustness of the conclusions.
4. Responsibilities of the independent statistician, the Interactive Web Response System (IWRS), and the Electronic Data Capture (EDC) system: Independent statistician (not involved in clinical efficacy assessments): (i) generates the random allocation sequence and maintains the complete randomisation schedule; (ii) performs unblinding only after database lock at study completion or following an authorised emergency unblinding review by the DSMB; (iii) is not involved in patient enrolment, efficacy evaluation, or safety adjudication. IWRS (Interactive Web Response System): (i) implements central randomisation to ensure allocation concealment; (ii) allows emergency unblinding requests via the system in urgent situations (requiring dual authorisation: request by the investigator and approval by the independent unblinding reviewer); (iii) maintains an audit trail for all randomisation operations. EDC (Electronic Data Capture): (i) collects all clinical efficacy and safety data; (ii) does not store any treatment allocation information within the EDC system, thereby ensuring that data entry personnel, clinical coordinators, and data managers remain blinded; (iii) treatment assignment codes are only imported by the independent statistician for analysis after database lock.

### 2.11 Intervention

#### 2.11.1 Basic information of the investigational drug

The investigational drug is SXYX, provided as granules. Each bag contains 10.0 g of granules. The granules are manufactured by the Department of Pharmacy (Hospital Preparation Unit), The First Affiliated Hospital of Henan University of Chinese Medicine. The Department of Pharmacy is qualified to prepare hospital-formulated herbal products under Good Manufacturing Practice-like conditions. Store at room temperature (15 °C–25 °C) in a dry and well-ventilated place, protected from light.

#### 2.11.2 Drug packaging and labeling

A label will be attached to the packaging of the investigational drug, displaying the following information: drug number, drug name, specification, dosage and administration, lot number, expiration date, precautions, storage conditions, production unit, and the statement “For clinical trial use only”. The packaging and labeling are designed to maintain blinding and randomization; the label will show only the drug number and will not reveal the patient's treatment allocation (SXYX or placebo).

#### 2.11.3 Treatment regimen

##### 2.11.3.1 Background therapy

Standard Western medical treatment will be provided according to the *2024 Chinese Guidelines for the Diagnosis and Treatment of Heart Failure* (28), and a set of principles for medication management and adjustment will be formulated.

(1) Principle of stable baseline medication
  Before randomisation, all subjects are required to undergo a stabilisation period of at least 2–4 weeks on guideline-directed medical therapy (GDMT), and the medication regimen at the end of this stabilisation period is defined as the “baseline treatment regimen”. Criteria for medication stability are: (i) no arbitrary change in drug class; (ii) the dosage of any individual drug should not fluctuate by more than 25% relative to the recent stable regimen.
(2) Principles for adjustment of drug class and dosage
  Throughout the trial, investigators are strongly encouraged to maintain the baseline medication regimen unchanged. If the initiation of any new core heart failure therapy, particularly sodium–glucose co-transporter 2 inhibitors (SGLT2i), angiotensin receptor–neprilysin inhibitors (ARNI), and mineralocorticoid receptor antagonists (MRA), becomes necessary due to newly emerging serious clinical indications, approval must be obtained from the principal investigator (PI) of the participating centre, and the event must be documented as a major protocol deviation, which will be excluded in sensitivity analyses performed in the final statistical analysis. Loop diuretic doses may be adjusted at any time to manage volume status; however, if the diuretic dose increases by >50% from baseline and persists for more than 72 h, this will be classified as a “major medication adjustment”. For medications used to treat comorbidities, the drug class and dosage should remain unchanged in the absence of safety concerns; if adjustment is truly necessary, it should be strictly in accordance with clinical guidelines, and the adjustment should be recorded.
(3) Criteria for initiation of rescue therapy
  To ensure patient safety while maintaining the integrity of the modified intention-to-treat (mITT) analysis set, rescue therapy should be initiated if any of the following criteria are met: (i) worsening signs or symptoms of heart failure requiring temporary intravenous diuretic use or hospitalisation for heart failure; (ii) an increase in loop diuretic dose of ≥30% from baseline at two consecutive visits, or a single dose up-titration of ≥100%; (iii) sustained weight gain of ≥2 kg within 7 days after increasing the oral diuretic dose; (iiii) symptomatic hypotension (systolic blood pressure <90 mmHg) or a decline in estimated glomerular filtration rate (eGFR) of >30% from baseline, induced by standard pharmacotherapy.
(4) Documentation standards for medication adjustments
  At each study visit (screening, baseline, weeks 4, 8, 12, 18, and 24), all concomitant medications and any adjustments must be recorded completely and in detail in the case report form (CRF). The documentation should include the medication name, dose, frequency, start and end dates of administration, and the reason for the adjustment.
(5) Pre-specified subgroup stratification and statistical adjustment strategy
  Randomisation stratification factor: baseline use of sodium–glucose co-transporter 2 inhibitors (SGLT2i) will be added as a mandatory stratification factor at the randomisation stage (stratified as “used” vs. “not used”). Subgroup analyses: pre-specified subgroup analyses will be performed to examine the treatment effect of the study intervention on peak oxygen consumption (peak VO_2_), according to baseline exposure to SGLT2i and glucagon-like peptide-1 receptor agonists (GLP-1 RAs). Statistical adjustment plan: to control for residual confounding, major medication adjustments and cumulative diuretic exposure (uniformly converted to furosemide equivalent doses) will be included as time-varying covariates in the mixed-effects model for the primary endpoint. In addition, a per-protocol sensitivity analysis will be conducted, excluding subjects with major protocol violations.

##### 2.11.3.2 SXYX group

On the basis of background therapy, patients will additionally receive SXYX Granule, taken with warm water after a 0.5-hour interval following Western medications. The dosage is one bag per dose, three times daily (morning, midday, and evening). The treatment duration is 24 weeks.

##### 2.11.3.3 Placebo group

On the basis of background therapy, patients will additionally receive a placebo matching SXYX Granule. The administration method and treatment duration are the same as those in the SXYX group.

### 2.12 Investigational drug management

The study team will initially distribute the investigational drug to each center according to its expected enrollment schedule and record the distribution. Subsequent shipments will be dynamically adjusted based on actual enrollment progress, and an inventory alert threshold will be set (e.g., when the stock falls below a 2-week supply) to prevent drug shortages. In this double-blind trial, the site drug administrator at each study center will be responsible for drug dispensing and patient instruction on proper use. The drug packaging and labeling comply with blinding and randomization requirements, and measures will be taken to ensure the drug does not deteriorate during storage and transportation. After the trial is completed, the site drug administrator will collect and count the remaining drug and return it to the study team.

Medication adherence will be calculated as (actual dose taken ÷ expected dose) × 100%. Adherence between 80% and 120% is considered good. The actual dose taken is calculated as: dispensed amount − returned amount − lost amount. For patients with poor adherence, the reasons should be identified and documented.

### 2.13 Visit schedule and assessments

At Visit 1 and Visit 7, the following will be recorded: patient demographics, medical history, concomitant medications, vital signs, physical examination, as well as assessments including cardiopulmonary exercise testing (CPET), N-terminal pro-brain natriuretic peptide (NT-proBNP), echocardiography, 6MWT, NYHA functional class, EQ-5D score, TCM syndrome score, coagulation function, routine blood, urine, and stool tests, urine pregnancy test, liver and kidney function tests, electrocardiogram (ECG), chest CT (only at Visit 1), and others. At Visit 2, randomization numbers will be assigned, the investigational drug or placebo will be randomly dispensed, and concomitant medications will be recorded. At Visit 1 and Visit 5, the following assessments will be performed: CPET, NT-proBNP, echocardiography, coagulation function, urine pregnancy test, and chest CT (only at Visit 1). At Visit 1, Visit 3, and Visit 5, the following assessments will be performed: 6MWT, NYHA functional class, EQ-5D score, TCM syndrome score, routine blood, urine, and stool tests, liver and kidney function tests, electrocardiogram (ECG), and others. At Visit 1, Visit 3, Visit 4, Visit 5, and Visit 6, major symptoms and signs will be recorded. Adverse events (AEs) and serious adverse events (SAEs) will be monitored and recorded throughout the trial. See Table 3 for the visit schedule and assessments.

**Table 3:** Visit schedule and assessments.

Assessment items	Study phase	Screening period	Enrollment period	Treatment period				
	Visit	1	2	3	4	5	6	7
	Week	-1W	0W	4W ± 3D	8W ± 3D	12W ± 3D	18W ± 3D	24W ± 3D
Screening of participants		√						
Signing of informed consent		√						
Medical interview	General information	√						
	Medical history	√						
	Concomitant medications	√	√	√	√	√	√	√
	Vital signs	√		√		√		√
	Physical examination	√		√		√		√
Efficacy measures	Peak VO_2_	√				√		√
	NT-proBNP	√		√		√		√
	Echocardiography	√				√		√
	NYHA class	√		√		√		√
	6MWT	√		√		√		√
	TCM syndrome scale	√		√		√		√
	Major symptoms/signs	√		√	√	√	√	√
	EQ-5D score	√		√		√		√
	Coagulation function	√				√		√
Safety measures	Routine blood test	√		√		√		√
	Routine urinalysis	√		√		√		√
	Routine stool test+occult blood^a^	√		√		√		√
	Liver function test	√		√		√		√
	Kidney function test	√		√		√		√
	Urine pregnancy test	√				√		
	Electrocardiogram (ECG)	√		√		√		√
	Chest CT	√						
Wearable monitoring devices			√	√	√	√	√	√
Multi-omics analysis		√						√
Inclusion criteria		√						
Exclusion criteria		√						
Screening number		√						
Randomization number assignment			√					
Dispensing of study drug and diary card			√	√	√	√	√	
Return of study drug and packaging				√	√	√	√	√
Return of diary card				√	√	√	√	√
Overall assessment	Adherence assessment			√	√	√	√	√
	Adverse event reporting and follow-up		√	√	√	√	√	√
	Efficacy assessment			√	√	√	√	√

a Perform stool routine and occult blood tests at Visits 1, 3, 5, and 7 when a bowel movement occurs or when the stool color is found to be black.

### 2.14 Outcome measures

#### 2.14.1 Primary outcome

Change from baseline to 24 weeks in peak VO_2_ as assessed by CPET.

#### 2.14.1.1 Standard operating procedure (SOP) for cardiopulmonary exercise testing (CPET)

Based on the references (29, 30), the following detailed rules have been formulated.

1. Standardised CPET protocol and equipment calibration: All participating centres will employ a computer-controlled, individualised ramp protocol, with the duration of each test controlled within 8–12 min. A cycle ergometer is preferred as the testing device; if treadmill testing is used, strict calibration of speed and grade increments is required. To ensure the integrity and reliability of the data, biochemical calibration of the gas analysers and flow sensors is performed before each test, using standard calibration gas mixtures and a 3-L calibration syringe.
2. Standardised criteria for maximal exertion and data processing: A test is deemed a maximal exercise test only if the participant meets at least one of the following criteria: peak respiratory exchange ratio (RER) ≥1.10, and/or heart rate reaching ≥85% of the age-predicted maximal heart rate. Peak oxygen consumption (peak VO_2_) is calculated using a 30-second rolling average of breath-by-breath data obtained during the final phase of exercise.
3. Pre-test medication management and standardised operator training: To minimise the interference of medications with metabolic measurements, and in the absence of clinical contraindications, the following washout intervals are required before CPET: at least 4 half-lives after the last dose of SGLT2 inhibitors and diuretics; and at least 24 h after the last dose of beta-blockers and other guideline-directed medical therapy (GDMT) agents. In addition, investigators and technicians at all participating centres are required to complete a standardised CPET qualification and training assessment.
4. Central blinded adjudication: To ensure reproducibility across multiple centres, 10% of the de-identified original CPET datasets from each participating centre will be randomly selected by blinded core laboratory personnel for blinded centralised adjudication.
5. Optimised reporting system for primary endpoint measures: In addition to body-weight-adjusted peak VO_2_ (mL/kg/min), this study will report two supplementary measures: absolute peak VO_2_ (mL/min) and percentage of predicted peak VO_2_ (%), calculated using the Wasserman/FRIEND reference equations.

#### 2.14.2 Secondary outcomes

1. Change from baseline to 12 weeks in peak VO_2_ as assessed by CPET.
2. NT-proBNP.
3. Echocardiographic parameters: Cardiac structure and function assessed by two-dimensional echocardiography using the Simpson method, including left ventricular end-diastolic diameter (LVEDD), LVEF, global longitudinal strain (GLS), left atrial strain, E/A ratio, E/e‘ ratio, E-wave deceleration time (DT), left atrial volume index (LAVI), pulmonary artery systolic pressure (PASP), interventricular septal thickness, left ventricular free wall thickness, and left ventricular mass index (LVMI).
4. New York Heart Association (NYHA) functional class.
5. 6MWT.
6. TCM syndrome scale: Qi deficiency with blood stasis syndrome score.
7. Major symptoms and signs: dyspnea, palpitations, chest tightness, shortness of breath, fatigue, etc.
8. EQ-5D score.
9. Coagulation function.

#### 2.14.3 Safety outcomes

1. Routine blood, urine, and stool tests, electrocardiogram (ECG), liver and kidney function tests.
2. Urine pregnancy test (for women of childbearing potential).
3. Chest CT.
4. Adverse events (AEs) or serious adverse events (SAEs).

### 2.15 Data collection and management

1. eCRF completion: Data must be sourced from original documents. All data must be entered in a timely, accurate, complete, standardized, and truthful manner. No items should be left blank. Any corrections must be documented with the reason for change.
2. eCRF review: The clinical research coordinator (CRC) will promptly complete, review, and submit the eCRF, and respond to queries raised by the data manager or clinical research associate (CRA). After data cleaning, the principal investigator will sign to confirm the data.
3. EDC database: A study-specific EDC database will be designed. User acceptance testing (UAT) will be completed before the enrollment of the first patient. All users must complete training before accessing the database.
4. Data entry and verification: Data will be promptly entered and submitted after each visit. The data manager and medical reviewer will review the data and issue queries. After the investigator responds to the queries, the data will be confirmed.
5. Medical coding: Adverse events will be coded using the latest Chinese version of the Medical Dictionary for Regulatory Activities (MedDRA). Concomitant medications will be coded using the second edition of the *Dictionary of Contemporary Drug Trade Names and Aliases*.
6. Blind review and unblinding: After all data tasks are completed, a blind review meeting will be convened to determine the analysis populations and lock the database. After the database is locked, the first unblinding will be performed, during which the statistician will be unblinded only to Group A versus Group B (without knowing which group is SXYX or placebo), followed by statistical analysis. At the final study meeting, the second unblinding will be performed with all participants (investigators, sponsor representatives, and the statistician) together, after which the study report will be signed and finalized.
7. Wearable Device Data Collection and Management
  1) Device information: Maiya Smart ECG Ring (Maiya Technology, China, model TK30). The device is equipped with a high-performance ECG sensor (GH3228), a PPG sensor (GPSE1602B), and an accelerometer (SC-7A20H), and is compatible with Android 9.0 and iOS 10.0 and above. 2) Pre-trial validation study: To ensure the reliability of data obtained from the wearable device in the target HFpEF population, a pre-trial validation study will be conducted before the main trial is initiated. A total of 32 patients who meet the trial entry criteria (8 from each sub-centre) will be enrolled. Each participant will undergo standard 12-lead electrocardiography (ECG) and pulse oximetry monitoring while wearing the device. Data will be collected under two conditions: (i) at rest (10 min in the supine position), and (ii) immediately after the 6-minute walk test (6MWT). The validation will assess: (i) heart rate accuracy: Bland–Altman analysis will be performed to calculate the mean bias and limits of agreement between the device-derived heart rate and that from the 12-lead ECG; the device will be considered acceptable if the mean absolute error for heart rate is ≤5 beats/min; (ii) oxygen saturation accuracy: the device-recorded SpO_2_ will be compared with that from pulse oximetry, with an acceptable mean absolute error of ≤2%; (iii) step count (distance): device-recorded data will be compared with the actual distance walked during the 6MWT, with an acceptable mean absolute error of ≤5%. 3) Wear time: Participants will wear the ring continuously for 1 week at baseline, week 12, and week 24. To ensure adequate data acquisition, at least 4 valid days are required at each assessment time point. A valid day is defined as a day with wear time ≥23 h (objectively verified by the device's accelerometer and skin-contact sensor) and ≥8 h of valid signal recording. 4) Data quality standards: Within each valid day, at least 8 h of valid ECG/PPG recordings (excluding artefact-contaminated segments) are required. If more than 30% of the recorded signal is classified as artefact (e.g., motion artefact, poor skin contact, or electrical interference), the data from that day will be excluded from analysis. All device-derived data will be reviewed by a blinded central core laboratory, and a random 10% sample will be subjected to additional manual quality checks to ensure inter-rater reliability. 5) Handling of missing data: Multiple imputation (MI) will be employed under the assumption of conditional missing at random (MAR), given the covariates included in the analysis model (baseline values, treatment allocation, visit time) and previous observed values. The imputation model will include all covariates used in the descriptive analysis model for the wearable device data, and will incorporate auxiliary variables that influence the missingness mechanism (e.g., signal quality flags, wear logs) to improve precision. Imputation will be performed only for measures that are complete at baseline but missing at subsequent visits (week 12 or week 24) due to <4 valid days; subjects with complete baseline data missing will not be included in the primary MI analysis, but will be retained in the modified intention-to-treat (mITT) set for other endpoint analyses. Twenty imputed datasets will be generated; the descriptive analysis model will be fitted to each, and the estimates and standard errors will be pooled using Rubin's rules. To explore the potential impact of the MAR assumption on the trend of results within an exploratory framework, a worst-case exploratory scenario analysis will be conducted: missing values in the intervention group will be replaced by the group observed mean +2 standard deviations (in the direction of worsening), and those in the control group by the group observed mean −2 standard deviations (in the direction of improvement). The results of this scenario analysis will be reported descriptively, comparing whether the direction and magnitude of the effect estimates are consistent with those of the primary analysis; all results will be clearly labelled as exploratory, with no formal statistical significance testing or adjustment for multiplicity. 6) Derived outcome measures: Mean heart rate, step count (distance), oxygen saturation, heart rate variability, and nocturnal sleep duration.

### 2.16 Study oversight and quality assurance

1. The study team will establish an expert steering committee to provide guidance on issues encountered during the study.
2. An investigator's study manual will be prepared, and relevant standard operating procedures (SOPs) will be developed to ensure the objectivity of clinical data collection.
3. Before the start of the study, all study personnel will receive training and undergo a consistency assessment.
4. A quality control (QC) team will be established. A quality monitor will periodically inspect the study conduct and hold regular meetings to discuss specific issues arising from the work.
5. A data management center will be established, and operational rules for data management will be formulated to ensure data accuracy.
6. Quality Control for Traditional Chinese Medicine (TCM) Syndrome Assessment
  1) Standardised training and assessment content: All investigators responsible for syndrome differentiation at each sub-centre will receive unified central remote training. Before formally participating in subject screening and enrolment, they are required to pass a test consisting of 10 standardised case scenarios, in which their syndrome differentiation and syndrome score results must achieve a Kappa coefficient and intraclass correlation coefficient (ICC) of ≥0.80 against the gold standard (established by two senior TCM attending physicians). 2) Discrepancy resolution mechanism: i) Internal preliminary review (at the sub-centre level): Each sub-centre must be staffed by at least two certified investigators (primary investigator and co-investigator). When the initial assessing investigator has doubts regarding the syndrome type or the assignment of syndrome scores, an immediate two-person independent (blinded) re-assessment must be initiated. If the two agree, the consensus is adopted; if they disagree and cannot reach a consensus after internal discussion, the case is escalated to secondary adjudication. ii) Central arbitration (at the expert panel level): For cases that cannot be resolved at the sub-centre level, the complete case documentation must be uploaded to the data management system within 24 h and submitted to the Central Adjudication Committee (composed of three senior TCM attending physicians not involved in the clinical trial at that centre). A majority voting rule (at least 2/3 of votes) will be applied for rapid adjudication. iii) Regular calibration and feedback: The Central Adjudication Committee will summarise all “disputed cases” and “adjudication outcomes” on a monthly basis, revise the Investigator's Manual accordingly, and provide targeted re-training to the relevant sub-centres, so as to reduce the occurrence of similar disputes at the source. 3) Blinded assessment: The Clinical Research Associate (CRA) will randomly select tongue-pulse photographs and syndrome scales from 5% of enrolled cases at each centre, and conduct a cross-centre retrospective consistency review. Should the Kappa coefficient and ICC at any sub-centre fall below the above thresholds, or should the rate of disputes be excessively high, we will suspend enrolment at that centre until re-training has been completed and the test has been passed.

### 2.17 Missing data and protocol deviation handling

For the primary endpoint, the MMRM provides valid estimates under the missing-at-random (MAR) assumption using all available observed data. To test the robustness of the MAR assumption, two sensitivity analyses will be performed: (i) complete-case analysis, restricted to patients who completed the full 24-week follow-up; and (ii) worst-case scenario analysis, in which missing values in the SXYX group are imputed with the minimum observed value and those in the placebo group with the maximum observed value. For secondary endpoints with a missing rate >10%, multiple imputation (MI) using a chained-equation approach will be applied, with the imputation model including all covariates and outcome variables. For patients who meet the mITT criteria (i.e., have received at least one dose of the study drug and have at least one post-baseline CPET assessment), if they experience any of the following events—(i) premature discontinuation of the study drug, (ii) receipt of rescue therapy for worsening heart failure (e.g., intravenous diuretics), (iii) hospitalisation for any reason, (iiii) Medication adjustments due to various reasons, or (iiiii) inability to complete CPET—they will still be retained in the mITT set (in accordance with the intention-to-treat principle), but will be excluded from the per-protocol set for sensitivity analyses. All protocol deviations will be summarised and listed by treatment group, and submitted to the Data Monitoring Committee for review in the final report.

### 2.18 Statistical analysis

Statistical analyses will be performed using SPSS Statistics 32.0 (IBM Corp., Armonk, NY, USA) and R 4.2 (for mixed models). Three analysis populations are defined: (i) the modified intention-to-treat (mITT) set, comprising all randomized patients who received at least one dose of the study drug and had at least one post-baseline primary efficacy assessment—this serves as the primary analysis set; (ii) the per-protocol set (PPS), including patients who completed the 24-week trial without major protocol deviations, used for sensitivity analyses; and (iii) the safety set (SS), including all patients who received at least one dose of the study drug, used for safety analyses. Baseline and descriptive analyses: Baseline demographic and clinical characteristics (including age, sex, BMI, aetiology of heart failure, LVEF, NT-proBNP, SGLT2i use rate, etc.) will be compared between the two groups using independent-samples *t*-tests for continuous variables with normal distribution, Mann–Whitney *U* tests for continuous variables with non-normal distribution, or chi-square tests for categorical variables, in order to describe the balance after randomization. For safety analyses, comparisons of the incidence of adverse events between groups will also be performed using chi-square tests or Fisher's exact test. Primary endpoint analysis: The change in peak VO_2_ from baseline to week 24 is designated as the sole primary efficacy endpoint (week 12 change is reclassified as a key secondary endpoint). The primary analysis will be performed using a mixed-effects model for repeated measures (MMRM) rather than ANCOVA, with fixed effects including treatment group, visit (baseline, week 12, week 24), treatment-by-visit interaction, baseline peak VO_2_, baseline SGLT2 inhibitor use (yes/no), and study center. The MMRM was chosen because it uses all available longitudinal data and accounts for the correlation between repeated measurements within each subject, whereas ANCOVA would be limited to a single post-baseline time point and would not adequately handle the repeated-measure structure. Subject is included as a random effect (random intercept). The treatment effect will be reported as adjusted least-squares mean difference with 95% confidence intervals. Secondary endpoint analysis: Key secondary endpoints (including change in peak VO_2_ at week 12, NT-proBNP, 6-minute walk test, and NYHA functional class) will be analyzed using the same MMRM for continuous variables or logistic regression for categorical variables (with NYHA class dichotomized as improvement ≥1 grade vs. no improvement). To control type I error inflation due to multiplicity, a fixed-sequence testing procedure is prespecified: formal hypothesis testing for these four key secondary endpoints will proceed only if the primary endpoint (week 24 peak VO_2_) is statistically significant (*P* < 0.05), in the following order: ① week 12 peak VO_2_ change → ② NT-proBNP → ③ 6MWT → ④ NYHA class improvement (≥1 grade). All other secondary endpoints (including echocardiographic parameters, TCM syndrome score, individual symptoms/signs, EQ-5D, and coagulation function) are designated as exploratory/supportive endpoints, for which only descriptive statistics and unadjusted *P*-values will be reported; between-group comparisons for these exploratory endpoints will be performed using *t*-tests or Mann–Whitney *U* tests as appropriate, with unadjusted *P*-values provided for descriptive reference only, explicitly labelled as hypothesis-generating and not subject to formal inferential conclusions; hence, no multiplicity adjustment is applied to them. Center heterogeneity: Study center is included as a fixed effect in all MMRM models to account for potential variations in CPET execution, background care, and baseline characteristics across sites, ensuring robustness of the pooled treatment estimate.

## 3 Discussion

Globally, the incidence, prevalence, hospitalization rate, and non-cardiovascular mortality of HFpEF are all higher than those of HFrEF, making it the predominant form of heart failure in the globally aging population and imposing a substantial disease and economic burden on society (31–33). Although modern medicine has made notable progress in the treatment of HFpEF, the underlying mechanisms remain incompletely understood. To date, only caloric restriction, exercise training, SGLT2 inhibitors, and dual SGLT1/2 inhibitors have demonstrated clinical benefit (31). HFpEF is a highly complex clinical syndrome often associated with multiple comorbidities such as diabetes, hypertension, and atrial fibrillation. More than 95% of patients with HFpEF have two or more such comorbidities (6). Therefore, single-target pharmacological therapy in modern medicine often fails to achieve optimal outcomes, leaving the clinical needs of HFpEF patients unmet. TCM emphasizes treatment based on syndrome differentiation. The theory of “treating different diseases with the same method” (Yì Bìng Tóng Zhì), which means that different diseases can be treated with the same herbal formula based on the same underlying syndrome, is a unique strength of TCM and is particularly well-suited for managing complex clinical syndromes such as HFpEF.

The composition of SXYX follows a rigorous principle of herb compatibility. The sovereign herbs, raw *Hedysarum Multijugum Maxim*, *Codonopsis Radix*, and *Cimicifugae Rhizoma*, supplement Qi and elevate sunken Qi, and carry the effects of the formula upward. The minister herbs, *Radix Paeoniae Rubra*, *Curcumae Rhizoma*, *Trichosanthes Kirilowii Maxim*, *Allium Azureum Ledeb*, *Arum Ternatum Thunb*, and *Radix Bupleuri*, activate blood circulation and resolve stasis, as well as eliminate phlegm and remove turbidity. The assistant herbs, *Cinnamomi Ramulus* and *Lepidii Semen Descurainiae Semen*, unblock Yang, transform Qi, and promote diuresis; *Anemarrhenae Rhizoma* clears heat and nourishes Yin, counterbalancing the warm-dry nature of the Qi-supplementing herbs. The messenger herb, *licorice*, harmonizes the actions of the various ingredients and protects the middle burner (spleen and stomach). The overall effect of the formula is to supplement Qi and activate blood circulation, making it particularly suitable for treating HFpEF with the syndrome of Qi deficiency and blood stasis. HFpEF is secondary to comorbidity-driven systemic inflammation, progressive fibrosis, and myocardial cell injury (31). Preclinical evidence has suggested that flavonoids derived from *Astragalus membranaceus* inhibit Th1 and Th17 cell differentiation and increase regulatory T cell (Treg) levels, thereby reducing inflammatory damage (34). It has also been shown in animal studies that astragaloside IV and *Astragalus* polysaccharide (APS) regulate the aberrant activation of the TGF-β1/Smads pathway, downregulate p38 MAPK and NF-κB/p65 phosphorylation levels, and inhibit myocardial fibrosis (35). Additionally, studies based on isolated compounds have indicated that total paeony glycoside (TPG) inhibits the activation of the NLRP3 inflammasome and alleviates the inflammatory response (36). Similarly, experimental evidence suggests that the β-sitosterol component of Gualou Xiebai Banxia Decoction reduces the levels of IL-1β, IL-6, and TNF-α in endothelial cells, thereby inhibiting inflammatory responses, cardiomyocyte apoptosis, and myocardial fibrosis (37, 38). Preclinical data have also demonstrated that cinnamaldehyde exerts anti-inflammatory effects by modulating multiple signaling pathways, including TLR4, TRAF6, NF-κB, JAK/STAT, and Nrf2, through the regulation of inflammatory cytokines such as TNF-α, IL-1β, and IFN-γ (39). Furthermore, findings from rodent models suggest that the aqueous extract of *Lepidii Semen Descurainiae Semen* (Tinglizi) activates the PI3K/Akt/mTOR signaling pathway, inhibits apoptosis, and ameliorates ventricular remodeling in rats with heart failure (40). Finally, one preclinical study has shown that Shengxian Yixin Granule inhibits myocardial fibrosis, delays ventricular remodeling, and improves cardiac function in rats with myocardial infarction via the PI3K/AKT signaling pathway (41). Taken together, it may be hypothesized that SXYX could exert its therapeutic effects on HFpEF through multiple pathways, including the inhibition of inflammatory responses, myocardial fibrosis, and apoptosis. However, it is important to note that the above mechanistic evidence is primarily derived from individual herbs, isolated compounds, or animal models; these findings should be interpreted as providing biological plausibility rather than proof of the mechanism of action of the complete SXYX formula in patients with HFpEF. Confirmation of these mechanistic hypotheses in the current human trial is warranted.

Patients with HFpEF typically present with dyspnea and reduced exercise tolerance as their classic clinical features. CPET assesses dyspnea and gas exchange during exercise, and serves as an important tool for assisting in the diagnosis and risk stratification of HFpEF (42). CPET is a safe, non-invasive, incremental-load exercise test. During CPET, peak VO_2_ refers to the highest oxygen consumption achieved during maximal exercise. This parameter reflects the metabolic status of HFpEF patients and is also an important prognostic indicator in patients with chronic heart failure (CHF) (43). In the early stages of the disease, NT-proBNP levels and their dynamic changes are of great prognostic value in predicting long-term outcomes in patients with HFpEF (44). Myocardial fibrosis is one of the key pathological features of HFpEF. NT-proBNP levels are associated with markers of myocardial fibrosis in HFpEF and are associated with an increased risk of adverse events (45). Therefore, using CPET-derived parameters and NT-proBNP as efficacy measures to evaluate the therapeutic effects and clinical prognosis of SXYX in patients with HFpEF is of significant clinical value.

The major innovation of this study lies in the following aspect: Given that traditional cardiopulmonary exercise testing can only be performed in a hospital setting and cannot reflect patients’ daily exercise tolerance, this study overcomes the limitations of the traditional single-modality assessment of exercise tolerance in HFpEF patients by introducing wearable monitoring devices to achieve out-of-hospital, real-time, long-term, and dynamic capture of patients’ cardiopulmonary exercise data, including heart rate, blood oxygen saturation (SpO_2_), step count, walking distance, and other parameters, thereby establishing a comprehensive evaluation model consisting of in-hospital standardized assessment plus out-of-hospital long-term dynamic monitoring. This model will evaluate the clinical efficacy of SXYX in treating HFpEF from multiple dimensions. Meanwhile, the protocol has been designed in strict accordance with the SPIRIT 2025 guidelines to ensure the generation of high quality clinical evidence. In summary, this study will provide an objective, pragmatic, and patient centred evaluation of the therapeutic efficacy of SXYX in a real world setting, thereby offering high level evidence to support their clinical application. Future research should focus on exploring the integration of wearable-derived digital biomarkers into routine clinical decision-making for HFpEF.

## Ethics statement

This study was approved by the Ethics Committee of The First Affiliated Hospital of Henan University of Chinese Medicine on 14 February 2026 (approval No. 2026HL-041-01) and was registered with the ITMCTR under registration number ITMCTR2026000833. The participants provided their written informed consent to participate in this study.
